# From Reading to Healing: Teaching Medical Professionalism through Literature

**DOI:** 10.5195/jmla.2020.827

**Published:** 2020-01-01

**Authors:** Eleanor Shanklin Truex

**Affiliations:** Medical Librarian, Chicago Metro Region for AMITA Health Saint Joseph Hospital-Chicago, AMITA Health Saint Francis Hospital-Evanston, and AMITA Health Saints Mary & Elizabeth Medical Center-Chicago, IL, etruex62@gmail.com

Susan Stagno and Michael Blackie’s book on fostering medical professionalism via close reading of classic and pop literature is a gem of a book. This well-organized anthology describes a variety of pedagogic scenarios designed to explore the concept of professionalism that can be incorporated into medical school or any health sciences curriculum. As aptly stated in the foreword by Arthur Frank:

This volume collects what was diffuse, thus showing the breadth of expertise that now exists in teaching health humanities, both in medical schools and undergraduate programs. This book is a collegial: teachers tell us what has worked for their classes, offering experienced guidance but inviting further diversity and innovation. (p. xi)

Stagno and Blackie are quite clear as to the need for this book, extolling the virtues and power of stories in our lives. In her introduction, Stagno comments on the clinical side of medical professionalism and the benefits of learning it:

Stories help us deal with chaos, ambiguity and complexity. These concepts are important in the learning of and teaching about professionalism because professionalism is much more than following the rules, being nice, or reciting principle-based ethical concepts” (p. xvi).

I cannot think of a better description of the state of modern health care and the need we have as members of that field to hold to our humanity and stories as health care and medicine become increasingly more technology driven and technology reliant. She continues:

Readers of literature can quite easily, I have found, put themselves in the situation of characters in the story, whether they are the doctor or others, even if the story is set in distant Siberia more than one hundred years ago. And being immersed in that situation, while also hearing the perspectives of other learners who might see the situation quite differently, they can be curious, question, evaluate, react emotionally—any of a variety of responses—and explore the margins of what it means to be a doctor, nurse, healthcare professional, or just a human being. (p. xvi–xvii)

In his introduction, Blackie covers the rationales regarding this mode of teaching, which is helpful to readers who are nonprofessional teachers. Straightforward lectures are not enough and appear to not engage the students; role models and mentors may not consistently live the qualities they espouse; teachable moments may be few and far between and, upon occasion, missed. According to Blackie:

“These concepts [altruism, duty, truthfulness, and empathy, to name a few] speak to ways of being—of professing—in the practice of medicine, especially when faced with situations rife with ambiguity. For example, educators have noted an association between an intolerance of ambiguity and declines in medical students’ attitudes toward underserved, geriatric, alcoholic, and chronic-pain patients. Such intolerance for ambiguity challenges professionalism at its core. Providing students with opportunities to engage with ambiguity in literary representations during their education prepares them for its appearance in clinical and other healthcare settings. (p. xx)

The book is laid out in a logical, systematic way. Following the introductions (which can be skipped entirely, if you prefer to skip forewords and introductions), a few reprints of foundational articles are collected in part I, “The Humanities in Medical Education: Some Key Texts.” These should not be skipped, because they provide the framework on which to build the lesson elements. They also depict, over the course of part I, the development of this pedagogy in undergraduate medical education. Pivotal to this entire approach is the concept, explained by Jane Gallop, in “Ethics in Reading,” of *close reading*, a method of “looking at what is actually on the page, reading the text itself, rather than some idea ‘behind the text.’ It means noticing things *in the writing*, things in the writing that stand out” (p. 6). This way of analyzing any literature (and film, described in subsequent sections by other contributors) is the primary tool of Stagno and Blackie’s premise.

Several contributors rightly reiterate that our basic education regarding reading almost anything written is to derive the gist of the piece, summarize the main points, and move on. In contrast, close reading demands the reader pay attention to nuance: phrasing, vocabulary, imagery, punctuation, even grammar. The reader must also read for content; in other words, the close reader must note the methods of the writer to convey their message—and the actual message—and see how one either fosters the other or hinders it.

This is a painstaking way (and a viable one) to create familiarity with any text and, therefore, the derivation of deeper meaning from the author’s work. But it is also a method for training medical or any students to pay attention to details and critically evaluate those details, a skill vital to all in the health care professions.

Once the foundation is understood, the other six sections—”Boundaries,” “Empathy and Respect,” “Authority and Duty,” “Stigma,” “Truth-Telling and Communication,” and finally, “Conclusion: Cautionary Words”—cover various aspects of professionalism and how they have been taught by a variety of professors at universities across the United States and Canada using classical literature, popular fiction, short stories, and film. When possible, the shorter pieces are included in this anthology, making access easier for readers. Each contributor delineates the specific literary pieces they chose to demonstrate that section’s focus—whether in a seminar, a workshop, small groups, or reading aloud—often with commentary on the student reception of these lessons.

Peppered throughout are bits of practical advice and appendixes that provide reading guides. Not just fiction is chosen: there are pieces here from medical journals, such as the *Annals of Internal Medicine*, and physicians who are also authors are both referred to and used as content in some programs. Being familiar with some of the chosen works (such as the film *Ordinary People* or the novel *Prince of Tides*), I found these discussions of the lesson plans fascinating and of general interest. The one weakness I perceived in the book is the lack of mention of social media and its fragmenting and isolating effects on health care professionals and patients alike. Perhaps a future edition will include discussion of the contributions of medical blogs and, more largely, social media as both a source for and an obstacle to stories.

For comparison purposes, Colin Robertson’s *Storytelling in Medicine: How Narrative Can Improve Practice* (CRC Press, 2017) is similar in terms of subject but more limited in scope. The two overlap in intention—to bring the humanities to medical education and practice—but not in focus. Robertson’s work deals with patient narratives as well as student narratives, with two sections of the nine in the book dealing with the use of stories in medical education. Stagno and Blackie’s anthology is more comprehensive and can be used to establish a humanities curriculum, whereas Robertson’s would likely be more useful for a specific class in medical humanities.

*From Reading to Healing* is sure to be used as reference book by medical school faculty and could benefit nursing and other health services education programs. The need for professionalism is a constant, no matter the amount of technology that modern medicine produces, so is the need for empathy and critical thinking.

**Eleanor Shanklin Truex, BSN, RN, MLIS,** etruex62@gmail.com, Medical Librarian, Chicago Metro Region for AMITA Health Saint Joseph Hospital-Chicago, AMITA Health Saint Francis Hospital-Evanston, and AMITA Health Saints Mary & Elizabeth Medical Center-Chicago, IL

